# Improved Diver Communication System by Combining Optical and Electromagnetic Trackers

**DOI:** 10.3390/s20185084

**Published:** 2020-09-07

**Authors:** Aman Kataria, Smarajit Ghosh, Vinod Karar, Takshi Gupta, Kathiravan Srinivasan, Yuh-Chung Hu

**Affiliations:** 1 Thapar Institute of Engineering and Technology, Patiala 147004, India; ammankataria@gmail.com (A.K.); smarajit67@gmail.com (S.G.); 2 Chief Scientist, CSIR-CSIO, Chandigarh 160030, India; vkarar@rediffmail.com; 3 Information Security Engineering, Soonchunhyang University, Asan-si 31538, Korea; takshi_gupta2012@hotmail.com; 4 School of Information Technology and Engineering, Vellore Institute of Technology (VIT), Vellore 632 014, India; kathiravan.srinivasan@vit.ac.in; 5 Department of Mechanical and Electromechanical Engineering, National ILan University, No. 1, Sec. 1, Shennong Rd., ILan 26041, Taiwan

**Keywords:** aquatic communication, electromagnetics, optics, tracking, underwater communication

## Abstract

The increasing need for observation in seawater or ocean monitoring systems has ignited a considerable amount of interest and the necessity for enabling advancements in technology for underwater wireless tracking and underwater sensor networks for wireless communication. This type of communication can also play an important role in investigating ecological changes in the sea or ocean-like climate change, monitoring of biogeochemical, biological, and evolutionary changes. This can help in controlling and maintaining the production facilities of outer underwater grid blasting by deploying unmanned underwater vehicles (UUVs). Underwater tracking-based wireless networks can also help in maintaining communication between ships and divers, submarines, and between multiple divers. At present, the underwater acoustic communication system is unable to provide the data rate required to monitor and investigate the aquatic environment for various industrial applications like oil facilities or underwater grit blasting. To meet this challenge, an optical and magnetic tracking-based wireless communication system has been proposed as an effective alternative. Either optical or magnetic tracking-based wireless communication can be opted for according to the requirement of the potential application in sea or ocean. However, the hybrid version of optical and wireless tracking-based wireless communication can also be deployed to reduce the latency and improve the data rate for effective communication. It is concluded from the discussion that high data rate optical, magnetic or hybrid mode of wireless communication can be a feasible solution in applications like UUV-to-UUV and networks of aquatic sensors. The range of the proposed wireless communication can be extended using the concept of multihop.

## 1. Introduction

In recent years, the inclination towards optical and magnetic tracking-based wireless communication has increased in the fields of terrestrial, underwater, and space links due to its low power and provision of high data rates [1,2]. Considerable research has been carried out for space and terrestrial links, but less emphasis has been placed on underwater communication as it is a more tedious and challenging job compared to atmospheric links of communication [3]. For efficient underwater communication, there are a certain number of issues. The major issue is the type of underwater environmental conditions, which range from shallow coastal water to deep-sea or oceans [3]. Acoustic waves are commonly used for underwater communication whose performance is degraded by high losses in transmission and reception of signals, low bandwidth, high latency, and Doppler spread. All these drawbacks can cause temporal and spatial variation of the acoustic channel, which further limits the available bandwidth of the system [4]. According to the real-time water communication data, the current underwater acoustic communication can support up to tens of kbps of data rate for long distances (in km) and up to some hundreds of kbps standard for short-range (few meters) [5,6]. Acoustic communication is categorized in different links. Table 1 provides the typical bandwidth for different underwater acoustic communication links with various ranges [7].

To achieve effective communication between underwater vehicles and sensors, a high data rate (up to few tens of Mbps) is required. Fiber optics or copper cables can be employed to get a high data rate, but they have several engineering and maintenance issues. Therefore, to overcome this problem a wireless link with a high data rate is an appropriate alternative. Two different wireless communication technologies, optical and magnetic tracking-based wireless communication techniques have been discussed in this paper. Based on the performance of optical and magnetic trackers, the hybrid version has also been proposed for better efficiency and low latency in communication. Optical tracking-based communication has an advantage of high bandwidth, but it can be severely affected by factors like dispersion, scattering, fluctuations in temperature, and beam steering. Moreover, the line of sight (LOS) is the prime necessity in optical tracking-based wireless communication. Due to these limitations, optical tracking based wireless communication is limited to short distances. However, optical trackers with blue-green sources and detectors are quite effective despite some limitations under seawater. Up to a few hundred meters, an optical tracker with blue-green sources is capable of providing underwater communication. A summary of different optical tracking with different power of Light emitting Diode (LED) and Laser is presented in Table 2.

The other tracking-based wireless communication is electromagnetic (EM) technology to obtain a high data rate but for short-range communication. The data rate in electromagnetic tracking-based wireless communication depends upon the speed of electromagnetic waves which further depends on permeability (µ), permittivity (ε), conductivity (σ), and volume discharge density (ρ) which varies on the type of underwater conditions. Radio-frequency waves are highly attenuated by seawater, but electromagnetic tracking-based wireless communication is capable of providing an excellent communication link for a short range (up to 40–50 m) [16,17]. Tracking has many applications using different techniques [18,19]. The placement of transmitters for tracking can vary from different devices that can be drones or other Internet of Things (IoT) devices [20]. The tracking can also be done through Unmanned Aerial Vehicles (UAV) to track the IoT devices [21].

## 2. Related Work

M. Doniec et al. discussed optical tracking based on underwater communication. Their work was limited to the pool water in which the optical tracker faces fewer constraints like dispersion and scattering. Moreover, due to the indoor pool, the efficiency of optical communication was not degraded by sunlight interference [22]. L.I. Johnson et al. discussed the recent advancements in optical tracking-based communication. They did not mention other alternatives like acoustic and magnetic tracking based on underwater wireless communication [23]. Hu and Fei discussed the hybrid version of underwater acoustic-optical tracker-based wireless communication. The study carried by the authors consisted of some problems related to the underwater environment which were not discussed in detail [24]. S. Chen et al. discussed an underwater communication system based on optical links for the divers. Their system was limited to the short-range (about 20 m). The discussion of other tracking technologies to increase the range was lacking in their study [25]. Frater et al. discussed electromagnetic tracking based on underwater wireless communication for autonomous underwater vehicles (AUVs). They compared their study with acoustic tracking-based communication. The study of optical tracking-based wireless communication, which is better than acoustic-based communication in some aspects, was missing [26].

## 3. Optical Tracking-Based Model for Aquatic Wireless Communication

**Figure 1:**
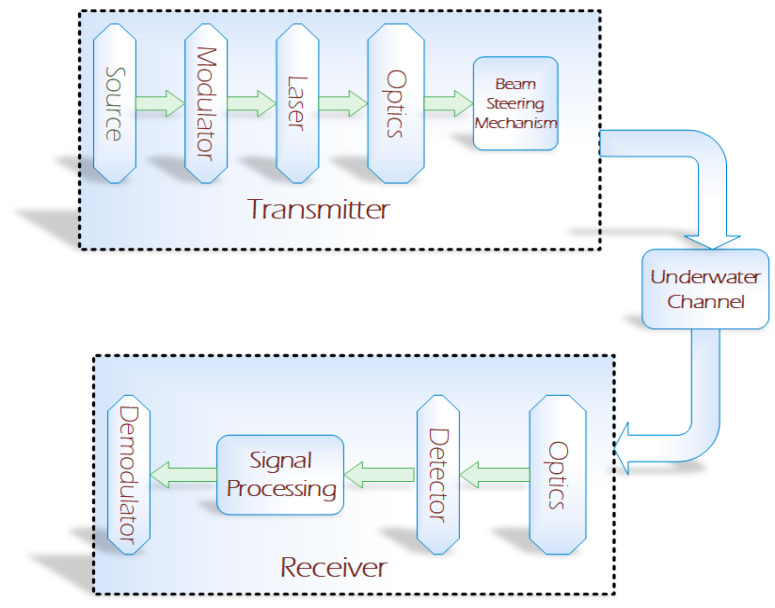
Block diagram of underwater optical communication.

Optical tracking has many applications, which vary from the human body (head, arm, leg, etc.) tracking to object tracking. These applications are mainly associated with a non-aquatic environment (outside the sea or ocean) [10]. In this work, the possibility of object tracking in the aquatic environment has been discussed. Figure 1 shows the basic block diagram of underwater optical communication [8]. The setup comprises a modulator and laser source. The laser beam is controlled by a beam steering mechanism. The transmitted signal is received at the receiver side and the output is produced at the receiver side after demodulation of the received signal.

However, due to suspended particles underwater, optical signals face many challenges as they lead to scattering of the optical signals. Disturbance in optical communication can also be caused due to sunlight. Line of sight is also the limitation of optical tracking [8]. Characteristics of different water bodies are not similar (varying from shallow water bodies to deep ocean). Their properties also vary geographically. Therefore, instead of deploying a single optical beam in an optical tracker, the diffused transmitted light is preferred to provide the broader underwater communication channel between transmitter and receiver.

For seawater, the water absorption coefficient is dependent on the chlorophyll concentration of the water. According to [14] the absorption coefficient of the seawater dependent on the chlorophyll concentration can be given in Equation (1) as:

(1) $$a\left(\lambda \right)=a_{pw}+a_{0}^{c}\left(\lambda \right)\;{\left(\frac{C_{c}}{C_{c}^{0}}\right)}^{0.602}+a_{f}^{0}C_{f}e^{\left(-k_{f}\lambda \right)}+a_{h}^{0}C_{h}e^{\left(-k_{f}\lambda \right)},$$

where $a_{pw}$ is the absorption coefficient of pure water in inverse meters, $a_{0}^{c}\left(\lambda \right)$ is the specific absorption coefficient of chlorophyll in inverse meters, λ is the vacuum length of light in nanometres, $C_{c}$ is the total concentration of chlorophyll which is in milligrams per cubic meter, $C_{c}^{0}$ = 1 mg/m^3^, $a_{f}^{0}$ is the specific absorption coefficient of fulvic acid which has the typical value of 35.959 m^2^/mg and is the first component of dissolved organic matter in the seawater, $k_{f}$ is the constant, having a value of 0.0189 nm^−1^, $a_{h}^{0}$ is the specific absorption coefficient of humic acid which has the typical value of 18.828 m^2^/mg and is the second component of dissolved organic matter in seawater, $k_{h}$ is the constant having value of 0.01105 nm^−1^, $C_{h}$ and $C_{f}$ are concentrations of humic and fulvic acids, respectively, in mg/cm^3^. The value of $a_{0}^{c}\left(\lambda \right)$ and $a_{pw}$ can be referred to from [6,7].

The chlorophyll profile $C_{CHL}$ over a depth *d*(*m*) from the surface can be described as a Gaussian curve that includes five parameters is given by Equation (2) [11]:

(2) $$C_{CHL}=B_{o}+S_{z}+\frac{h}{\sigma \sqrt{2\pi }}\;e^{\frac{-{\left(d-d_{max}\right)}^{2}}{2\sigma^{2}}},$$

where $S_{z}$ is the vertical gradient of the concentration, which is a negative value due to the slow decrement in chlorophyll concentration with the increasing depth of the seawater, d is the total concentration of chlorophyll above the background levels, and $d_{max}$ is the depth of the deep chlorophyll maximum. The standard deviation of chlorophyll concentration $\sigma_{CHLC}$ can be obtained using Equation (3) [27]:

(3) $$\sigma_{CHLC}=\frac{h}{\sqrt{2\pi \left[C_{CHL}\left(d_{max}\right)-B_{o}-S_{Z_{max}}\right]}},$$

**Figure 2:**
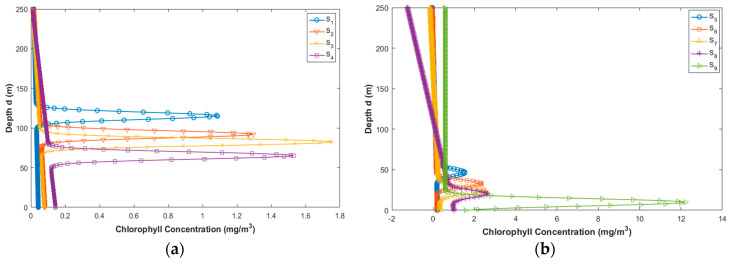
(**a**) Chlorophyll concentration depth profile different ranges of surface chlorophyll level S_1_–S_4_ [27]; (**b**) chlorophyll concentration depth profile for different ranges of surface chlorophyll level S_5_–S_9_ [27].

The chlorophyll concentration profiles differ in the ocean and, therefore, the profile shape alters. The ocean locations were allocated to one of the nine groups, and each group represented a different range of surface chlorophyll concentrations. The values of the chlorophyll concentrations are given in Table 3 below. Figure 2a,b represent the plot of chlorophyll profiles for S_1_–S_4_ and S_5_–S_9_ respectively.

**Figure 3:**
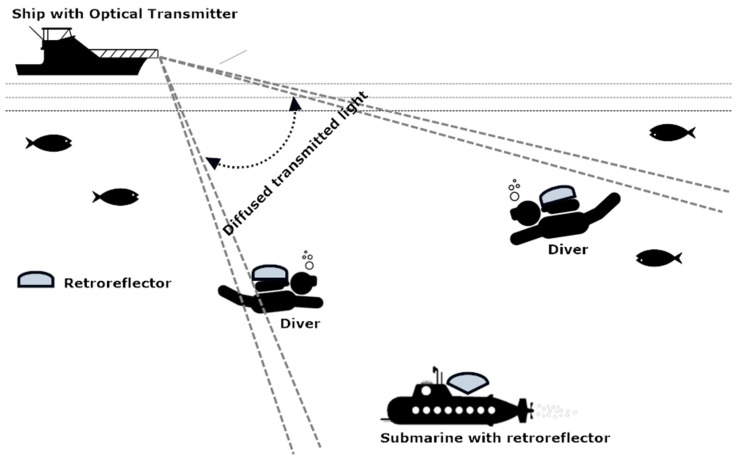
Optical tracking-based aquatic communication model.

The value of scattering and diffuse coefficient is low in the seawater. Therefore, the propagation of the optical beam is almost a straight line. Moreover, the attenuation caused by sunlight also plays an important role in optical tracker-based communication [29]. As we are considering pure sea or clear ocean for underwater communication, it is evident that the operating wavelength is 450–500 nm (blue-green region of visible light) [8]. For this purpose, an optical tracker with the transmitter using an argon ion laser or doubled Ti–sapphire can be used. A color-selective retroreflector link can be employed in the receiver to receive and reflect the blue-green laser to the transmitting source [30]. The $P_{RR}$ the received power through the retroreflective link can be given by Equation (4) [31]:

(4) $$P_{RR}=P_{T}\alpha_{T}\alpha_{R}\alpha_{R}l_{P}\left(\lambda \frac{d}{cos\Theta }\right)x\frac{a_{RR}cos\Theta }{2\pi x^{2}\left(1-cos\Theta_{d}\right)}\left(\frac{a_{R}cos\Theta }{\pi {\left(xtan\Theta_{R}\right)}^{2}}\right),$$

where $\alpha_{R}$ is the optical efficiency of the retroreflector, $a_{RR}$ is the aperture area of the retroreflector, $\Theta_{R}$ is the divergence angle of the retroreflector, $P_{T}$ is the average power of the optical transmitter, $\alpha_{T}$ is the optical efficiency of the transmitter, Θ is the angle which is perpendicular to the trajectory of transmitter-receiver and receiver plane, $a_{R}$ is the aperture area of the receiver, x is the perpendicular distance between receiver and transmitter plane. λ is the operating wavelength (450 to 500 nm in this case). Figure 3 presents the model for optical tracking-based aquatic communication.

## 4. Electromagnetic Tracking-Based Model for Wireless Aquatic Communication

**Figure 4:**
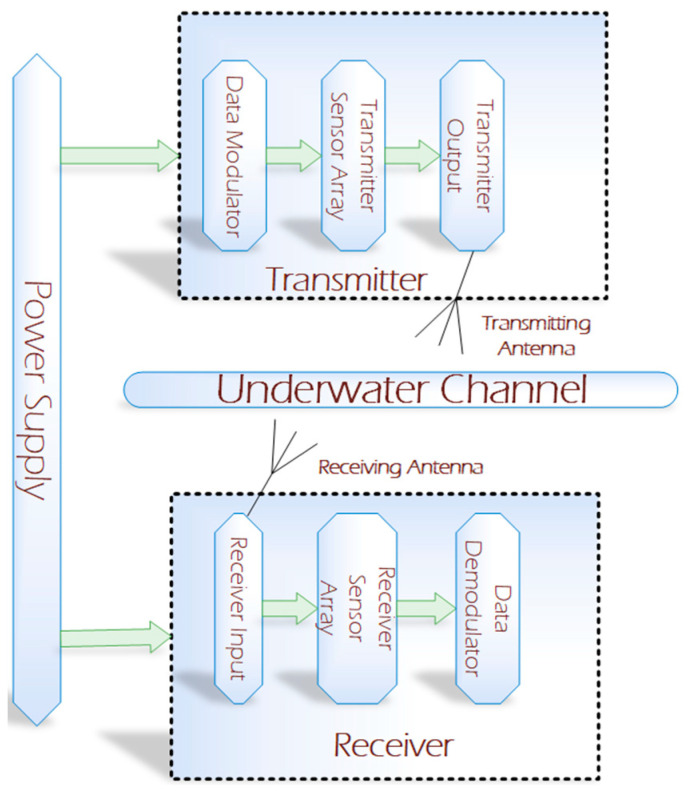
Block diagram of underwater electromagnetic communication.

Electromagnetic tracking can also be used for communication between underwater and terrestrial bodies. Electromagnetic tracking can be used for communication between a diver, deeply merged submarine, and the ship on the surface of the water [32]. The electromagnetic tracking waves can operate from 100 Hz to 10 Mhz. The range is the main limitation of electromagnetic tracking-based wireless communication as it is more efficient in the short-range [33]. However, communication cannot be hindered by limitations like a line of sight, scattering, dispersion, and sunlight. Electromagnetic waves can cross water or the sea bed easily. Figure 4 represents the basic block diagram of underwater electromagnetic communication [34]. The transmitter module consists of a data modulator and an array of sensors. The transmission of the signal is undertaken using the transmitting antenna. The receiving antenna receives the signal from the transmitter module and further processes it to the demodulation module for its final output [35].

The network used for tracking can be based on network communications [36]. It can also communicate without the need for LOS and is also more immune to acoustic noise [37]. The bandwidth of electromagnetic tracking-based wireless communication is very high (up to 100 Mbps) in a short-range. The propagation of electromagnetic waves in water is a challenging task as compared to air. This is due to the high electrical conductivity and permittivity of water. This problem can also be solved using a multihop approach [38,39]. The velocity of the electromagnetic wave underwater can be given by the general Equation (5) [40]:

(5) $$v=\sqrt{\frac{f\times 10^{7}}{\sigma }},$$

where σ is the conduction in water, f is the operating frequency, and v is the propagation velocity of the electromagnetic wave in the water. The wavelength λ is given by Equation (6) [40]:

(6) $$\lambda =\;\frac{1}{\sqrt{f\times \sigma \times 10^{-7}}},$$

$\delta_{skin}$ is the skin depth of the water which is given by Equation (7) [40]:

(7) $$\delta_{skin}=\frac{1}{2\pi \sqrt{f\times \sigma \times 10^{-7}}}.$$

**Figure 5:**
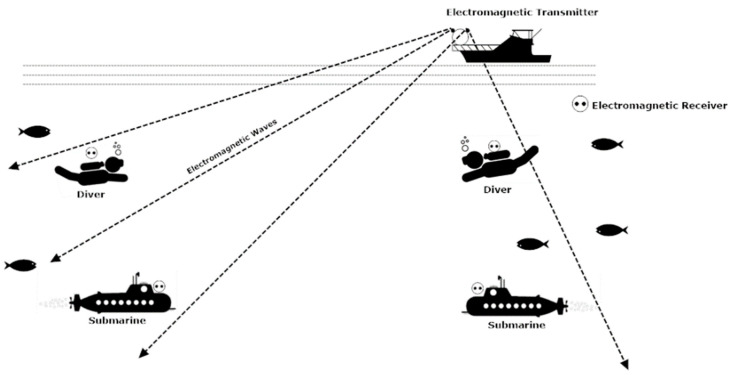
Electromagnetic tracking-based wireless communication.

Figure 5 shows the basic model of underwater electromagnetic tracking based on wireless communication.

Underwater, the conductivity σ is set to 3.2 S/m at 100 Hz frequency and 5.4 S/m at 10 MHz. Table 4 shows the typical values of the propagation velocity and wavelength (Equations (5) and (6) of an electromagnetic wave in seawater. For the sake of comparison, the propagation velocity of the acoustic tracking system is also presented, which is fixed at 1500 m/s.

**Figure 6:**
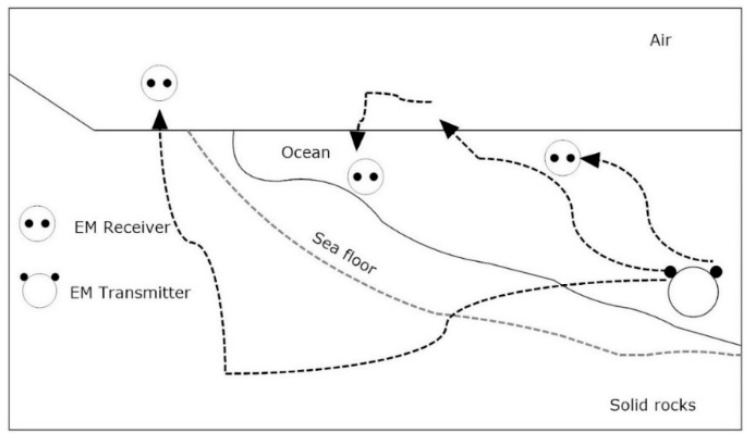
Multipath electromagnetic propagation in seawater.

It can be observed that the propagation velocity of the electromagnetic wave of 10 MHz frequency underwater is increased 100 times that of the acoustic tracking system. This is an important ingredient to control the latency. The impact of change in frequency on wavelength can also be observed in Table 4. As the frequency increases in seawater, the wavelength decreases in magnitude. This effect can help in the implementation of the navigational and sensing applications. It is also an interesting fact that electromagnetic waves get attenuated more in water than air due to which localized communication becomes easier in a multiuser environment. The effect of water to air interface is also an important consideration in electromagnetic tracking performance. Due to specific refraction loss and propagation loss, the electromagnetic wave crosses the water-to-air boundary and radiates from the small section of water directly above the transmitting end. Due to high permittivity and large refraction angle the signal travels almost parallel to the surface of the seawater. This phenomenon eliminates the need for surface repeater buoys in case of communication between land and a submerged station or a submarine. These all factors can enable multipath propagation of electromagnetic waves as shown in Figure 6 [40]. Table 5 represents the different data rates and applications of electromagnetic waves in water.

### 4.1. Absorption Coefficient in Seawater

**Figure 7:**
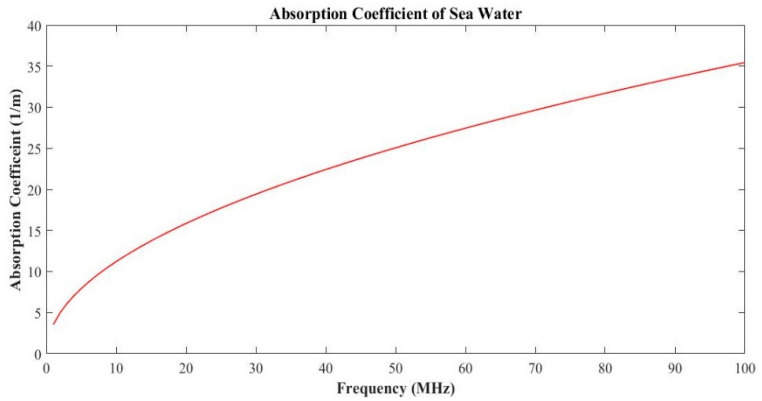
The absorption coefficient of seawater using electromagnetic wireless communication.

It is evident that the absorption coefficient α of an EM wave is dependent on the frequency of the wave. It is given by Equation (8) [13]:

(8) $$\alpha \approx \sqrt{\mu \sigma f\pi },$$

where μ is magnetic permeability, f is electromagnetic frequency, and σ is electrical conductivity. The absorption coefficient in the seawater is directly proportional to the electromagnetic frequency. However, as shown in Figure 7, the absorption coefficient increases with the increase in frequency, it becomes nearly impossible to communicate under the water using electromagnetic technology. Therefore, low frequency is preferred for electromagnetic tracking. Using low-frequency electromagnetic tracking can be achieved up to 25 m. This limitation can be eradicated by using a hybrid version of optical and magnetic tracking under the water, which is discussed in the next section.

### 4.2. Absorption Coefficient in Freshwater

The absorption coefficient α of an EM wave is independent of the frequency of the wave in freshwater. It is given by Equation (9) [13]:

(9) $$\alpha \approx \frac{\sigma }{2}\sqrt{\frac{\mu }{\epsilon }},$$

where σ is electric conductivity, μ is magnetic permeability and ϵ is dielectric permittivity. The rate of communication is high in the freshwater as the absorption coefficient is not dependent on frequency. It can be noticed in Equation (6) that the absorption coefficient will almost be the constant value. Therefore, electromagnetic tracking-based communication is more efficient in freshwater as compared to seawater.

### 4.3. The Velocity of Electromagnetic Tracking-Based Communication in Water

**Figure 8:**
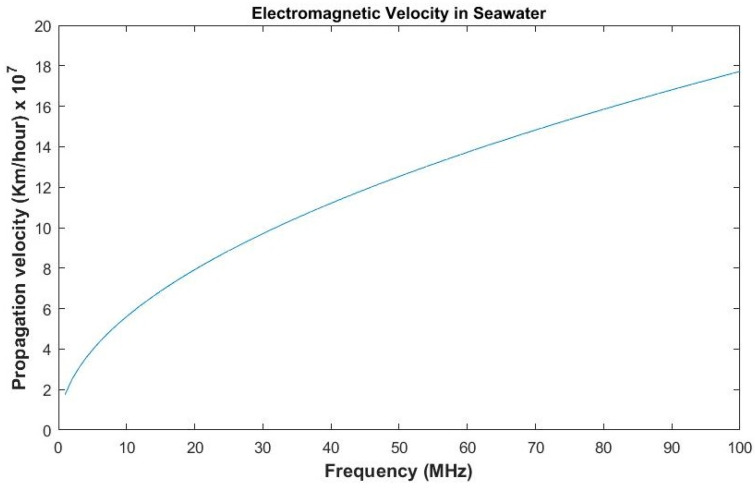
Electromagnetic wave velocity in seawater.

The propagation velocity of electromagnetic waves in seawater is frequency-dependent. It increases as the frequency value of electromagnetic waves is increased. Its value is approximated as given by Equation (10) [40]:

(10) $$V_{Pr}\approx \sqrt{\frac{4\pi f}{\sigma \mu }},$$

where $V_{Pr}$ is the propagation velocity of an electromagnetic wave in seawater, f is the frequency of an electromagnetic wave, σ is the conductivity, and μ is the permeability of an electromagnetic wave. However, the velocity of electromagnetic waves in freshwater is decreased by nine times and is almost constant as compared to the speed in air. The propagation velocity versus frequency is plotted in Figure 8.

### 4.4. Path Loss in Electromagnetic Underwater Communication

For efficient deployment of electromagnetic tracking-based communication, the channel characterization should be accurate. Channel path loss is the difference between the transmitted signal power and the received signal power. The power received by the receiver underwater is given by Equation (11):

(11) $$P_{r}=P_{t}+G_{T}-L_{P},$$

where $P_{r}$ power is received by the receiver (submarine/diver) under the water, $P_{t}$ is the power transmitted by the transmitter outside the water, $G_{T}$ is the total gain of receiver and transmitter, and $L_{P}$ is the free space path loss under the water in decibels. The efficiency of electromagnetic tracking-based communication is maximum in the range of 100 m. The $L_{P}$ is given by Equation (12):

(12) $$L_{P}=20\log_{10}\frac{4\pi d}{\lambda },$$

d is the distance between transmitter and receiver and λ is the wavelength in free space in meters. Table 6 represents the different types of tracking techniques used in underwater object tracking. The table also shows the single technique and hybrid techniques used for underwater object tracking. Optical and electromagnetic techniques have not been fused until now for underwater object tracking. Section 5 discusses the possible hybridization of optical and electromagnetic underwater tracking techniques.

## 5. Possible Prototype Model of a Hybrid Opto-Magnetic Tracking-Based Diver Tracking System

**Figure 9:**
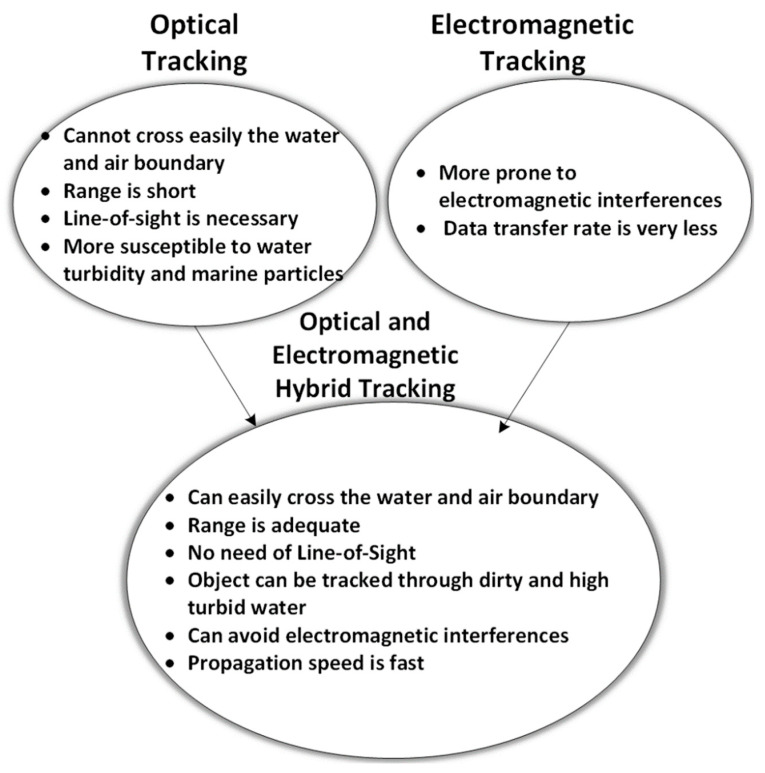
Feature of hybridized model of optical and electromagnetic underwater tracking.

There may be a lesser need for LOS in electromagnetic-based communication depending on the amount of obstruction present in the seawater. Moreover, there is no effect of scattering, sunlight, and much less effect of dispersion in electromagnetic waves as compared to optical-based tracking. On the other hand, optical technology needs LOS for communication. It is also affected by dispersion, scattering, and sunlight interference. However, its bit rate is much more than electromagnetic-based communication. Moreover, low power is required in optical communication. Therefore, taking advantage of both technologies, the hybrid version of both optical and electromagnetic tracking based wireless communication is discussed for aquatic communication. Figure 9 shows the features of the hybrid diver-tracking system.

In this manuscript, the amalgamation of optical and electromagnetic tracking is proposed for better efficiency of underwater diver tracking. Considering the optical tracking system for diver tracking, the power received by the receiver allows us to inspect the tracking range of an optical tracker in turbid harbor water with variation in distance. Using Equation (4) again, the power received $P_{RR}$ by the Optical retroreflector is given by:

(13) $$P_{RR}=P_{T}\alpha_{T}\alpha_{R}\alpha_{R}l_{P}\left(\lambda \frac{d}{cos\Theta }\right)x\frac{a_{RR}cos\Theta }{2\pi x^{2}\left(1-cos\Theta_{d}\right)}\left(\frac{a_{R}cos\Theta }{\pi {\left(xtan\Theta_{R}\right)}^{2}}\right),$$

The parameters used for simulating and studying the power received against different distances are given below in Table 7.

**Figure 10:**
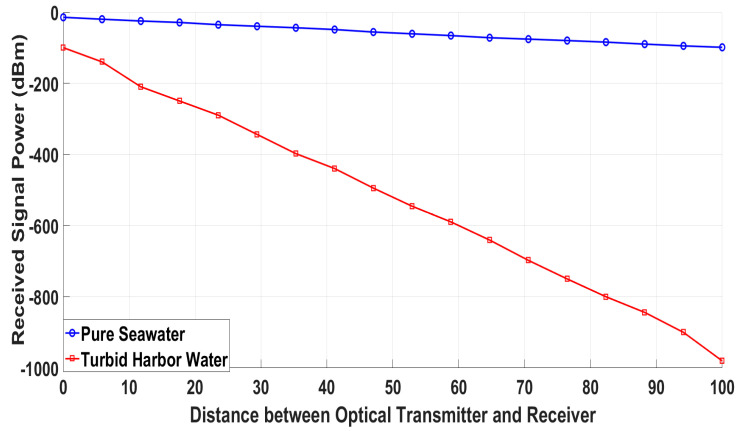
Signal power received by optical receiver against different distance value between diver and retroreflector.

The parameters in Table 7 are the general parameters in which the performance of an optical tracker is best. The transmitting power is generally chosen between 10 to 100 milliwatt for a tracking range of 100 m [8]. Divergence angle $\Theta_{Retro}$ is typically less than $\frac{\pi }{20}$ [31]. Transmitting and receiver efficiency lies between 0.4 and 0.9. Aperture area of the receiver is between 10 cm^2^ to 100 cm^2^ for optimum performance. The data rate is affected by aperture area of the receiver, optical depth range, and operating wavelength. Substituting the values of parameters given in Table 7 in the general equation of received power, Equation (4), for simulating the results. Figure 10 shows the simulation results of the optical tracker with different distances between diver and retroreflector in pure seawater and turbid harbor water.

**Figure 11:**
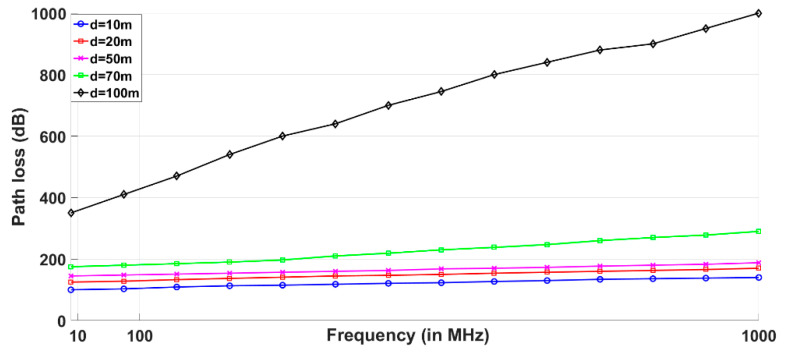
Path loss in electromagnetic tracking at different operational frequencies and distance between the diver and electromagnetic transmitter.

Now for a secondary component, the general path loss (in dB) in electromagnetic underwater tracking is given by Equation (14) [48].

(14) $$L=20\log\left(e^{\alpha d}\right),$$

where d is the distance between the diver and electromagnetic tracker (depth) and α is the propagation constant. Equation (13) is used for simulating and analyzing the path loss in electromagnetic tracking for different distances. Figure 11 shows the path loss in electromagnetic tracking against different distances between the diver and electromagnetic tracker. It is evident from Figure 11 that the path loss increases as a diver goes deep in the sea resulting in the inaccuracy of tracked coordinates. Coordinates can also be acquired in turbid water where chlorophyll matter is greater. This is the main advantage of an electromagnetic tracker.

**Figure 12:**
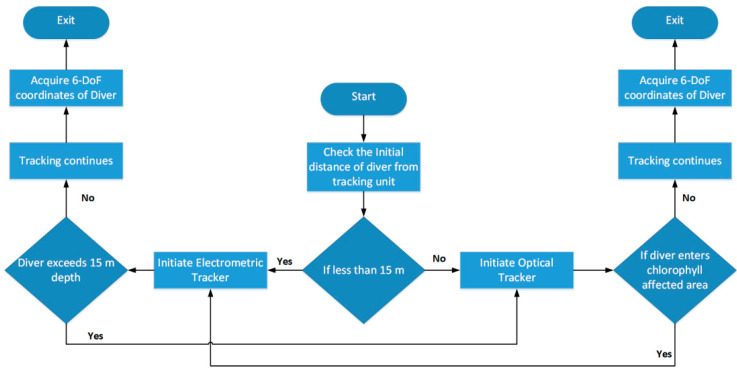
Flowchart of working of hybrid underwater diver tracking system.

The acquisition of 6-Degrees of Freedom (6-DoF) coordinates, that are X, Y, Z, Yaw, Pitch and Roll of the diver can be achieved through the hybrid tracking system by initiating it in the switching mode. The flowchart shown in Figure 12 describes the possible working of the hybrid diver-tracking system.

**Figure 13:**
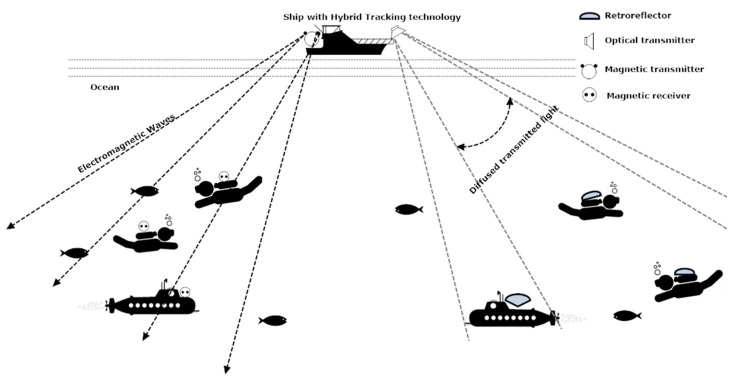
A hybrid model of optical and electromagnetic tracking-based aquatic communication.

The accuracy of an electromagnetic tracker is high up to 15–20 m, but after exceeding the depth of 20 m the accuracy of an electromagnetic tracker declines. The proposed flowchart is intended to initiate with electromagnetic tracking if the diver depth is less than 15 m. If the diver depth increases and the chlorophyll content is under the permissible level then an increase in path loss will switch the tracking to optical mode. Similarly, if chlorophyll content increases, then the decrease in received power signal will switch the tracking to optical mode. The purpose of the hybrid version is to increase the tracking accuracy of the 3-DoF axis (X, Y, and Z) of the objects underwater. Figure 13 shows the possible hybrid model of optical and electromagnetic tracking-based wireless communication in the sea.

**Figure 14:**
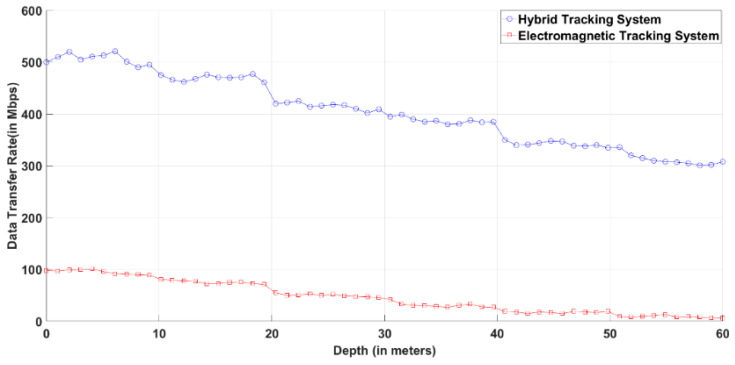
Data rate of the hybrid tracking system and electromagnetic tracking system.

Figure 14 represents the data rate of the electromagnetic tracker and hybrid tracker. The data rate decreases in the case of both electromagnetic tracker and hybrid tracker as the depth of the seawater increases due to turbidity and dirt in the seawater. However, the decrease in data transfer rate is significantly less in the case of a hybrid tracking system as compared to the electromagnetic tracking system alone. The data rate shown in Figure 14 is simulated using Equations (15) and (16).

The channel capacity of the electromagnetic tracker is given by Equation (15) [49].

(15) $$C_{erg}=E\left[\int_{0}^{B}log_{2}\left(1+\frac{S_{x}\left(f\right){\left|H_{r}\left(f\right)\right|}^{2}}{N_{0}}\right)df\right],$$

where $C_{erg}$ is ergodic capacity, $S_{x}\left(f\right)$ is the power spectrum density of the signal transmitted by the electromagnetic tracker, $N_{0}$ is the noise in the signal, $H_{r}\left(f\right)$ is the channel frequency response and E is the expectation. Data rate R in optical communication for both free space and underwater is given by the general Equation (16) [50].

(16) R=PT∈Ot∈OrAπ(θT2)2L2EpNb,

where $P_{T}$ is the power transmitted by the optical transmitter, $\in_{O_{t}}$ and $\in_{O_{r}}$ are the optical transmitter and receiver efficiency, respectively, A is the area of the receiving module, $\theta_{T}$ is the divergence angle of the transmitter, L is the range, $E_{p}=\frac{hc}{\lambda }$ is the energy of the photon, and $N_{b}$ is the sensitivity of the receiver (photons/bit).

## 6. Conclusions

In this article, we discussed the two dominant technologies for aquatic communication. Shortcomings of both the tracking technologies have been discussed in this paper. The optical tracking-based communication is severely affected primarily by sunlight interference, dispersion, and scattering. For this, it is evident that the optical wavelength in the blue-green region (450–500 nm) is most efficient for underwater communication. The alternative, which is electromagnetic-based underwater communication, is potentially effective for short-range. The propagation velocity of electromagnetic-based communication is much higher than the commonly used acoustic technology. Further, there is a lesser need for a line of sight in electromagnetic-based communication. To gain the benefits of both technologies, the possible hybrid model is discussed. The accuracy can be increased using a hybrid model.

## Figures and Tables

**Table 1:** The bandwidth of different acoustic communication links [5,6].

Distance	Range (km)	Bandwidth (kHz)	Data Rate
Very short	<0.1	>100	400–500 kbps
Short	0.1–1	25–60	≈35 kbps
Medium	1–10	≈15	≈15 kbps
Long	10–100	3–10	≈5 kbps
Very long	1000	<2	≈500–600 bps

**Table 2:** Different optical tracking sources used for underwater communication [8].

Distance (in Meters)	Power	Source	Data Rate	Ref.
20–30	550 mW	Blue LED	≈few Kbps	[9]
35–50	1 W	Laser	1 Gbps	[10]
200–250	5 W	LED	≈2 Mbps	[11]
≈5	40 mW	Laser	1.5 Gbps	[12]
≈5.5	15–20 mW	Laser	4.8 Gbps	[13]
31 (Deep sea)	0.1 W	LED	1 Gbps	[14]
30 (In pool)	5 W	LED	1.3 Mbps	[15]

**Table 3:** Values for S_1_–S_9_ chlorophyll concentration profiles [28].

	$C_{chl}\;(\mathrm{mg}/m^{3})$	$B_{o}\;(\mathrm{mg}/m^{3})$	$S\;x\;10^{-3}\;(\mathrm{mg}/m^{2})$	h (mg)	$d_{max}\;(m)$	$C_{chl}\;(d_{max})\;(\mathrm{mg}/m^{3})$	$d_{\infty }\;(m)$
S_1_	<0.04	0.0429	−0.103	11.87	115.4	0.708	415.5
S_2_	0.04–0.08	0.0805	−0.260	13.89	92.01	1.055	309.6
S_3_	0.08–0.12	0.0792	−0.280	19.08	82.36	1.485	282.2
S_4_	0.12–0.2	0.143	−0.539	15.95	65.28	1.326	264.2
S_5_	0.2–0.3	0.207	−1.03	15.35	46.61	1.557	200.7
S_6_	0.3–0.4	0.160	−0.705	24.72	33.03	3.323	226.8
S_7_	0.4–0.8	0.329	−1.94	25.21	24.59	3.816	169.1
S_8_	0.8–2.2	1.01	−9.03	20.31	20.38	4.556	111.5
S_9_	2.2–4	0.555	0	130.6	9.87	136.5	---

**Table 4:** Performance of electromagnetic waves in seawater [40].

Frequency	Propagation Velocity	Wavelength
100 Hz	1.77 × 10^4^	1.76 × 10^2^
10 MHz	4.30 × 10^6^	4.30 × 10^−1^
Acoustic	1.5 × 10^3^	-------

**Table 5:** Different data rates and applications of electromagnetic waves in water [5].

Range	Data Rate in Seawater	Possible Applications
<9 m	>9 kbps	Diver’s network
35–40 m	300–350 bps	Diver to Diver communication
150–200 m	15–20 bps	Underwater networking, Diver communication
>1.5 km	<1 b/s	Deep water communication

**Table 6:** Different techniques of underwater object tracking.

S.No	Author	Year	Tracking Technique Used	Sensors Used	Computational Technique Used	Hybrid Approach Used	Ref.
1	Lee et al.	2012	Visual-Based Tracking	Bowtech Divecam-550C	Underwater Color Restoration Algorithm	No	[41]
2	Mandic et al.	2016	Sonar Tracking	Soundmetrics ARIS 3000	Sonar Image Processing and Extended Kalman Filter	No	[42]
3	Magalhaes et al.	2013	Visual-Based Tracking	Sony Hyper Had, TS-6021PSC	Kanade–Lucas–Tomasi (KLT) Tracking	No	[43]
4	Chuang et al.	2016	Moving Camera-Based Tracking	A Combination of Trawl and Stereo-Camera System	Deformable Multiple Kernels Technique	No	[44]
5	Watson et al.	2014	Optical Tracking	Blue-Wavelength Gan Light Source Optical Sensor	Laser-based Tracking	No	[45]
6	Feezor et al.	1997	Electromagnetic Tracking	2 kHz Electromagnetic Transmitter	Autonomous Oceanographic Sampling Network (AOSN)	No	[46]
7	Dalberg et al.	2006	Acoustic and Electromagnetic Tracking	31 Element Hydrophone Array and 4 Hz low dipole	Kalman Filter	Yes	[47]

**Table 7:** Parameters used for simulating power received for different distances.

Parameter	Value
Transmitting Power	60 milliwatt
Transmitter Divergence Angle ($\Theta_{Retro}$)	1.6 mrad
Transmitter Efficiency ($\alpha_{Retro}$)	0.7
Receiver Efficiency	0.7
Optical Depth range for tracking	0 < d < 100 (in meters)
Aperture area of the Receiver	22.7 cm^2^
Operating Wavelength (λ)	475 nm
